# Early use of imipenem/cilastatin and vancomycin followed by de-escalation versus conventional antimicrobials without de-escalation for patients with hospital-acquired pneumonia in a medical ICU: a randomized clinical trial

**DOI:** 10.1186/cc11197

**Published:** 2012-02-15

**Authors:** Jong Wook Kim, Joowon Chung, Sang-Ho Choi, Hang Jea Jang, Sang-Bum Hong, Chae-Man Lim, Younsuck Koh

**Affiliations:** 1 Department of Gastroenterology, Asan Medical Center, University of Ulsan College of Medicine, 86 Asanbyeongwon-gil, Songpa-gu, Seoul 138-736, Korea; 2 Department of Pharmacy, Asan Medical Center, University of Ulsan College of Medicine, 86 Asanbyeongwon-gil, Songpa-gu, Seoul 138-736, Korea; 3 Department of Infectious Diseases, Asan Medical Center, University of Ulsan College of Medicine, 86 Asanbyeongwon-gil, Songpa-gu, Seoul 138-736, Korea; 4 Department of Pulmonary and Critical Care Medicine, Inje University Haeundae Paik Hospital, 1435, Jwa-dong, Haeundae-gu, Busan 612-030, Korea; 5 Department of Pulmonary and Critical Care Medicine, Asan Medical Center, University of Ulsan College of Medicine, 86 Asanbyeongwon-gil, Songpa-gu, Seoul 138-736, Korea

## Abstract

**Introduction:**

Although early use of broad-spectrum antimicrobials in critically ill patients may increase antimicrobial adequacy, uncontrolled use of these agents may select for more-resistant organisms. This study investigated the effects of early use of broad-spectrum antimicrobials in critically ill patients with hospital-acquired pneumonia.

**Methods:**

We compared the early use of broad-spectrum antimicrobials plus subsequent de-escalation (DE) with conventional antimicrobial treatment (non-de-escalation, NDE) in critically ill patients with hospital-acquired pneumonia (HAP). This open-label, randomized clinical trial was performed in patients in a tertiary-care center medical intensive care unit (MICU) in Korea. Patients (*n *= 54) randomized to the DE group received initial imipenem/cilastatin plus vancomycin with subsequent de-escalation according to culture results, whereas patients randomized to the NDE group (*n *= 55) received noncarbapenem, nonvancomycin empiric antimicrobials.

**Results:**

Between November 2004 and October 2006, 109 MICU patients with HAP were enrolled. Initial antimicrobial adequacy was significantly higher in the DE than in the NDE group for Gram-positive organisms (100% versus 14.3%; *P *< 0.001), but not for Gram-negative organisms (64.3% versus 85.7%; *P *= 0.190). Mean intensive care unit (ICU) stay, and 14-day, 28-day, and overall mortality rates did not differ in the two groups. Among culture-positive patients, mortality from methicillin-resistant *Staphylococcus aureus *(MRSA) pneumonia was higher in the DE group, even after early administration of vancomycin. Multidrug-resistant organisms, especially MRSA, were more likely to emerge in the DE group (adjusted hazard ratio for emergence of MRSA, 3.84; 95% confidence interval, 1.06 to 13.91).

**Conclusions:**

The therapeutic advantage of early administration of broad-spectrum antimicrobials, especially with vancomycin, was not evident in this study.

## Introduction

Early use of broad-spectrum antimicrobials and subsequent de-escalation (DE) after microbiologic culture results may minimize the emergence of drug-resistant organisms [1-3] and costs [1] during the treatment of patients with hospital-acquired pneumonia (HAP). This strategy may reduce the overall duration of antimicrobial treatment [4] and mortality rates [5,6]. These studies, however, were observational, with successful DE indicating less-resistant pathogens and/or less severity of infection.

The combination of carbapenem and vancomycin is frequently prescribed for the treatment of HAP, in which the prevalence of infection by multidrug-resistant (MDR) organisms is high. Although inadequate initial therapy may increase mortality rates [7], the frequent use of carbapenem plus vancomycin predisposes to the emergence of MDR organisms. Vancomycin may select for resistant pathogens once colonization takes place [8], and vancomycin use has been associated with increases in vancomycin-resistant enterococci (VRE) [9] and vancomycin-intermediate and -resistant *S. aureus *[10]. Treatment with carbapenems has also been reported to increase the overall risk for emergence of MDR organisms [11-13]. Moreover, the efficacy of vancomycin as empiric therapy for HAP in hospitals with a high prevalence of methicillin-resistant *Staphylococcus aureus *(MRSA) has not been completely addressed. Vancomycin is a relatively large molecule, with poor penetration into the alveolar lining and alveolar macrophages [14,15]. In addition, the cure rate for vancomycin in patients with MRSA pneumonia has been disappointing [16,17]. A study of febrile neutropenic patients with MRSA infection showed that these patients did not experience worse outcomes when vancomycin therapy was delayed until definitive diagnosis [18]. The use of carbapenem, which has the broadest spectrum of activity against Gram-negative organisms, must also be controlled to limit the selection and spread of MDR pathogens.

To examine these concerns, we conducted a randomized clinical trial in a large tertiary care center in Korea, where the methicillin resistance rate of *S. aureus *isolated from blood has been reported to be 72% [19]. In this trial, we compared the effects of early treatment with broad-spectrum antimicrobials followed by subsequent DE, with conventional antimicrobial regimens.

## Materials and methods

### Study design

This prospective, open-label, randomized clinical trial was performed in the 28-bed medical ICU of a 2,200-bed tertiary care hospital in Seoul, Korea (Asan Medical Center). Written informed consent was obtained from each patient or his or her legal guardian. This study was approved by the institutional review board at Asan Medical Center and was performed in accordance with the ethical standards laid down in the 1964 Declaration of Helsinki. From November 2004 to October 2006, patients were randomly assigned to receive either initial treatment with imipenem/cilastatin plus vancomycin, with de-escalation starting 3 to 5 days later, based on initial culture results (de-escalation group, DE), or conventional empiric noncarbapenem antimicrobial therapy (non-de-escalation group, NDE). The collected data are described in Additional file 1.

### Inclusion and exclusion criteria

Patients were eligible for inclusion if they were older than 18 years, had been hospitalized > 48 hours, and had been admitted to the ICU for treatment of established HAP. Patients were excluded if their pathogen(s) had been previously identified, if their antimicrobial regimen for HAP had been changed > 48 hours before ICU admission, if they were pregnant or lactating, or if they had HAP within 1 month before enrollment.

### Randomization

After eligibility had been confirmed and consent was given, patients were randomized 1:1 to the DE or NDE group, by using an allocation sequence based on a block size of four, generated by a computer random-number generator.

### Primary and secondary end points

The primary end point was initial antimicrobial adequacy. The secondary end points were mortality, emergence of MDR organisms, duration of antimicrobial treatment, and ICU stay. To compare the emergence of MDR organisms in the NDE and DE groups, patients with initially present MDR organisms were excluded.

### Sample size and statistical analysis

Based on a previous study [20], it was expected that the antimicrobial adequacy would be 48% for NDE and 94.2% for DE. To detect this difference, we calculated that inclusion of 60 patients per group was required (two-tailed α = 0.05; 80% power), allowing for 10% dropout. Recruitment period of 2 years was planned. We analyzed a modified intention-to-treat population. Categoric variables were compared by using the χ^2 ^test or the Fisher Exact test, and continuous variables were compared by using the Student *t *test. Cox proportional hazards models were used to calculate hazard ratios (HRs) and 95% confidence intervals (CIs) for the emergence of MDR organisms with and without de-escalation. To determine whether the proportional hazards assumptions held, we performed tests based on Schoenfeld residuals. All tests of significance were two-tailed, with *P *< 0.05 regarded as significant. Data were analyzed by using Stata version 11.1 (StataCorp, College Station, TX).

### Antimicrobial therapy and de-escalation policy

**Figure 1:**
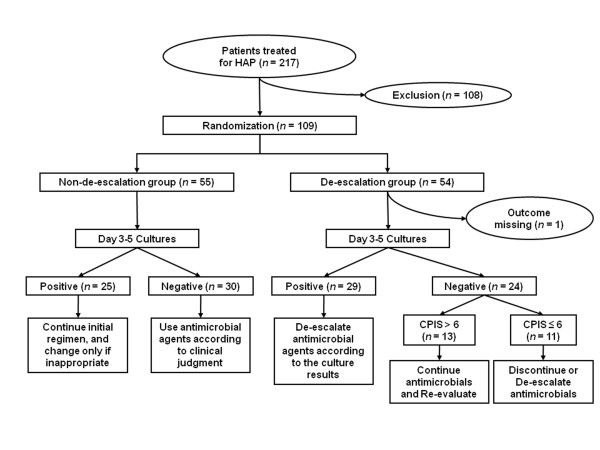
**Flow algorithm of treatment for patients with hospital-acquired pneumonia in this study**.

DE patients were treated with imipenem/cilastatin (0.5 g every 6 hours) and vancomycin (15 mg/kg every 12 hours), with dosages adjusted in patients with renal or hepatic functional impairment. After 3 to 5 days, imipenem/cilastatin and/or vancomycin were de-escalated individually, based on culture results and clinical status (Figure 1). If the organism(s) could not be identified (culture negative), the treatment was adjusted according to the day 3 to 5 clinical pulmonary infection score (CPIS) results. In culture-negative patients with a CPIS > 6 on day 3 of antimicrobial treatment, the initial antimicrobial treatment was not de-escalated, and the patient was re-evaluated for possible etiologic organisms. In culture-negative patients with CPIS of 6 or less, the initial antimicrobial treatment was either discontinued (vancomycin) or de-escalated (imipenem/cilastatin) [21].

NDE patients received initial antimicrobials empirically according to the American Thoracic Society (ATS)/Infectious Diseases Society of America (IDSA) guidelines for the management of adults with hospital-acquired, ventilator-associated, and healthcare-associated pneumonia [22], except that none received carbapenem antimicrobials or vancomycin, and no de-escalation was used. The initial choice of antimicrobials and all subsequent changes of regimens, due to antimicrobial inadequacy or treatment failure, were determined by the attending physician.

Discontinuation of antimicrobial treatment in both groups was recommended in the absence of purulent sputum, if WBC was < 10,000/mm^3 ^or decreased > 25% from its peak value, if body temperature was < 38.3°C, if improvement or lack of progression appeared on the chest radiograph, and if the PaO_2_/FiO_2 _ratio was > 250 mm Hg [4]. The usual durations of antimicrobial treatment were 7 days for non-MDR organisms, such as *S. aureus *and *H. influenzae*, and 8 to 14 days for MDR organisms such as *Pseudomonas aeruginosa*, *Acinetobacter baumannii*, and MRSA. If clinical improvement was not evident, the attending physician decided when to stop antimicrobial treatment [23].

### Definitions

HAP was diagnosed according to the American College of Chest Physicians criteria [22,24], defined as the occurrence of a new and persistent radiographic infiltrate occurring 48 hours or more after admission, in conjunction with two of the following: (a) fever (38.5°C or higher) or hypothermia (< 36.5°C); (b) leukocytosis (white blood cells > 10,000/mm^3 ^or < 4,000/mm^3^); or (c) purulent tracheal aspirate or sputum. Adequate antimicrobial treatment was defined as the administration of one or more antimicrobial agents with *in vitro *activity against the bacterial species associated with HAP [20]. Patients from whom pathogenic microorganisms could not be isolated from respiratory cultures were continued on their initial antimicrobial regimen [20]. These patients were considered not evaluable when determining antimicrobial adequacy and were excluded from the analysis. MDR organisms included MRSA; vancomycin-resistant enterococci; and certain Gram-negative bacilli, including those producing extended spectrum β-lactamase ESBLs; imipenem-resistant *Pseudomonas aeruginosa*; and organisms such as *Acinetobacter baumannii, Stenotrophomonas maltophilia, and Burkholderia cepacia*, which are intrinsically resistant to the broadest-spectrum antimicrobial agents [8]. Emergence of MDR organisms was defined as newly isolated MDR organisms during therapy, after excluding patients with MDR organisms isolated from initial cultures.

## Results

### Patients

During the 2-year study period, 217 patients were newly treated for HAP in our ICU. Of these, 108 patients were excluded because of previously identified pneumonia pathogens in 49, alterations in the antimicrobial regimen 48 hours or more before ICU admission in 36, refusal to participate in 18, and for other reasons in five. The remaining 109 patients were consecutively enrolled, with 108 included in our modified intention-to-treat analysis (Figure 1).

Table 1 shows a summary of baseline characteristics of the DE and NDE groups. Of the 109 patients, 88 were male (80.7%), and 21 were female (19.3%) patients. Their mean acute physiology and chronic health evaluation (APACHE) II score was 23.1 (standard deviation [SD], 5.6; range, 11 to 35). Other parameters did not differ significantly in these two groups. Adverse events that occurred during the study period are listed in Additional file 2.

**Table 1:** Baseline characteristics of patients in the DE and NDE groups

Characteristics	DE (*n *= 54)^a^	NDE (*n *= 55)	*P* ^b^
Gender			0.772
Male	43 (79.6%)	45 (81.8%)	
Female	11 (20.4%)	10 (18.2%)	
Age (range), year (IQR)	66.4 (61-77)	61.7 (51-71)	0.117
PaO_2_/FiO_2_, mean (SD)	181.8 (103.0)	177.7 (68.1)	0.813
Type of pneumonia			> 0.999^c^
Hospital-acquired pneumonia	50 (92.6%)	50 (90.9%)	
Ventilator-associated pneumonia	4 (7.4%)	5 (9.1%)	
On mechanical ventilation	47 (50.5%)	46 (49.5%)	0.449
Baseline APACHE II score, mean (SD)	23.3 (5.6)	22.8 (5.7)	0.607
Baseline SOFA score, mean (SD)	9.1 (3.1)	8.4 (3.6)	0.270
Baseline CPIS, mean (SD)	6.7 (1.4)	6.8 (1.3)	0.807
Prior hospital day (IQR)^d^	16.7 (4-17)	9 (4-10)	0.064
Prior ICU day (IQR)^d^	4.4 (1-5)	3.4 (1-5)	0.182
Prior ICU days > 5 days	33 (61.1%)	31 (56.4%)	0.416
Prior days on mechanical ventilation (IQR)^d^	3.3 (0-4)	2.5 (0-4)	0.347
Prior antimicrobial administration^e^	18 (34.6%)	24 (43.6%)	0.340
Initial presentation			0.943
Severe sepsis	27 (50%)	29 (52.7%)	
Septic shock	10 (18.5%)	10 (18.2%)	
ARDS	17 (31.5%)	16 (29.1%)	
Underlying disease			
Hypertension	14 (26.9%)	12 (22.6%)	0.611
Neurologic disease	15 (28.8%)	15 (28.3%)	0.951
COPD	5 (9.6%)	6 (11.3%)	> 0.999
Liver cirrhosis	5 (9.6%)	8 (15.1%)	0.555
CRF/ESRD	4 (7.7%)	3 (5.7%)	0.716^c^
Diabetes mellitus	16 (30.8%)	14 (26.4%)	0.621
Alcoholism	4 (7.7%)	4 (7.5%)	> 0.999^c^
Hematologic malignancy	1 (1.9%)	2 (3.8%)	> 0.999^c^
Modified McCabe score			0.968^c^
Life expectancy < 1 month	4 (7.5%)	2 (3.6%)	
Life expectancy 1 month to 5 years	40 (75.5%)	46 (83.6%)	
Life expectancy > 5 years	9 (17%)	7 (12.7%)	

Values given as mean (standard deviation) or number (percentage) of patients. ALI, acute lung injury; APACHE, acute physiology and chronic health evaluation; ARDS, acute respiratory distress syndrome; COPD, chronic obstructive pulmonary disease; CPIS, clinical pulmonary infection score; CRF, chronic renal failure; DE, de-escalation group; ESRD; end-stage renal disease; HAP, hospital-acquired pneumonia; IQR, interquartile range; NDE, non-de-escalation group; SOFA, sequential organ-failure assessment; VAP, ventilator-associated pneumonia. ^a^Clinical outcome of one patient in the DE group was not available. ^b^With the Student *t *test or the *χ^2 ^*test. ^c^With the Fisher Exact test. ^d^Days before study enrollment in mean (IQR), only including the time spent at Asan Medical Center (Seoul, Republic of Korea). ^d^Prior administration of antimicrobial agent within 2 days.

### Organisms identified at the initiation of the study

Initially isolated organisms in both study groups are shown in Additional file 3. Overall, 54 (50%) patients were culture positive. Culture methods included bronchoalveolar lavage in 37 patients, endotracheal aspirate in 62, expectorated sputum in 11, and blood culture in 20. No significant between-group differences were found in isolation rates of Gram-positive (*P *= 0.173) and Gram-negative (*P *= 0.920) organisms. Gram-positive organisms were more frequently isolated than were Gram-negative organisms (33.3% versus 26.9%), with MRSA being the single most frequent organism isolated from patients with HAP (*n *= 31; 28.7%).

### Initial antimicrobial agents and adequacy

The initial antimicrobial adequacy was significantly higher in the DE than in the NDE group (75.9% versus NDE, 48%; *P *= 0.035, Table 2). This was mostly due to the activity of vancomycin against Gram-positive organisms (*P *< 0.001 for Gram-positive and *P *= 0.190 for Gram-negative organisms). The most frequently prescribed antimicrobial combination in the NDE group was piperacillin/tazobactam plus ciprofloxacin (63.6%), followed by piperacillin/tazobactam plus aminoglycoside (20%), and ceftazidime plus either ciprofloxacin or aminoglycoside (9.1%).

**Table 2:** Number of patients who received adequate initial empiric antimicrobials in the DE and NDE groups^a^

Organism	DE	NDE	Total	*P * ^b^
		
	*n/m *(%)	*n/m *(%)	*n/m *(%)	
All patients	22/29 (75.9)	12/25 (48)	34/54 (63)	0.035
Gram-positive organisms	21/21 (100)	2/14 (14.3)	22/35 (65.7)	< 0.001
Gram-negative organisms	9/14 (64.3)	12/14 (85.7)	21/28 (75)	0.190

Values given as number (percentage) of patients. DE, de-escalation group; *m*, number of patients with isolated organisms; *n*, number of patients with adequate initial antimicrobial treatment; NDE, non-de-escalation group. ^a^Only culture-positive patients were included. ^b^With *χ^2^*or the Fisher Exact test.

### Rate of de-escalation in the DE group, and regimen change or escalation in the NDE group

We identified 36 patients in the DE group eligible for DE of vancomycin, and 33 eligible for DE of imipenem/cilastatin. Vancomycin was discontinued in 30 of these 36 patients (83.3%), whereas imipenem/cilastatin was de-escalated in 28 (84.8%) of 33, including DEs to piperacillin/tazobactam, with or without ciprofloxacin, in 17 patients, to ceftriaxone in five, to ceftazidime in three, to ampicillin/sulbactam in three, and to cefazolin in two. Of the patients in the NDE group, 18 underwent change of escalation of their antimicrobial regimen, either to carbapenem alone (*n *= 10) or to carbapenem plus vancomycin (*n *= 8).

### Treatment outcomes

The mean (SD) total duration of antimicrobial agents did not differ significantly in the DE and NDE groups (12.5 [5.8] days versus 14.1 [7.3] days; *P *= 0.222). In addition, no significant between-group differences were found in overall hospital mortality (44.2% versus 34.6%; *P *= 0.316), day-14 mortality (24.5% versus 13%; *P *= 0.314), and day-28 mortality (44.2% versus 25.9%; *P *= 0.131) rates (Table 3). The mean duration of ICU stay among survivors was longer in the DE than in the NDE group (21.1 versus 14.1 days), but the difference was not significant (*P *= 0.464). Hospital mortality rates did not differ significantly among patients with different types of pneumonia, being 40/99 (40.4%) for patients with HAP and three of nine (33.3%) in patients with ventilator-associated pneumonia (*P *> 0.999, data not shown). Overall, the mortality rates associated with Gram-positive organisms were higher than those associated with Gram-negative organisms (Table 4).

**Table 3:** Treatment outcomes of patients with HAP in the DE and NDE groups

	DE (*n *= 53)	NDE (*n *= 55)	Overall (*n *= 108)	*P*
Time to adequate antimicrobials, days, mean (SD)^a^	1.9 (0.5)	2.8 (0.6)	2.4 (0.4)	0.280
Mortality				
Day 14	13 (24.5%)	9 (16.7%)	22 (20.6%)	0.314
Day 28	21 (39.6%)	14 (25.9%)	35 (32.7%)	0.131
Hospital mortality	23 (44.2%)	18 (34.6%)	41 (39.4%)	0.316
ICU stay, days, mean (IQR)^b^	21.1 (6-35)	14.1 (6-19)	17.2 (6-19)	0.464^c^
Emergence of MDR organisms^d^	11 (37.9%)	7 (16.7%)	18 (25.4%)	0.043
Time to development, days, mean (IQR)	19.4 (11-30)	22.7 (9-30)	21 (11-30)	0.108^c^
Methicillin-resistant *S. aureus*	8 (27.6%)	4 (9.5%)	12 (16.9%)	0.059
Gram-negative non-Enterobacteriaceae	4 (13.8%)	5 (11.9%)	9 (12.9%)	> 0.999
*S. maltophilia*	3 (10.7%)	2 (4.8%)	5 (7.1%)	0.383
Imipenem-resistant *A. baumannii*	0	2 (4.8%)	2 (2.9%)	0.513
Imipenem-resistant *P. aeruginosa*	0	1 (2.4%)	1 (1.4%)	> 0.999
ESBL-producing *K. pneumonia*	1 (3.6%)	0	1 (2.9%)	0.400

Values given as number (%) of patients, unless otherwise stated. DE, de-escalation group; ESBL, extended spectrum β-lactamase; IQR, inter-quartile range; MDR, multidrug-resistant; NDE, non-de-escalation group; SD, standard deviation. ^a^Time from initial diagnosis of pneumonia to administration of adequate antimicrobial agent among initially culture-positive patients. ^b^Excluding patients who died. ^c^With the Kruskal-Wallis rank test. ^d^MDR organisms isolated within 1 month of study enrollment. Patients with initial MDR organisms were excluded (24 patients in the DE and 13 in the NDE group). MDR organisms include methicillin-resistant *S. aureus*, imipenem-resistant *P. aeruginosa*, imipenem-resistant *A. baumannii*, *S. maltophilia*, and extended-spectrum β-lactamase-producing organisms.

**Table 4:** Hospital mortality relative to initial antimicrobial adequacy

Adequacy	No. of deaths/episodes (%)	*P*
Overall (*n *= 54)		0.304
Inadequate	6/20 (30%)	
Adequate	15/34 (44.1%)	
Gram-positive organism (*n *= 35)		0.476
Inadequate	4/12 (33.3%)	
Adequate	12/23 (52.2%)	
Gram-negative bacilli (*n *= 28)		0.639
Inadequate	1/7 (14.3%)	
Adequate	6/21 (28.6%)	

Excluding culture-negative patients.

### Emergence of MDR organisms

In 18 (25.7%) patients, new MDR organisms appeared within 1 month of antimicrobial treatment, at a mean of 21 days (range, 9 to 30 days). The overall rate of emergence of MDR organisms was significantly higher in the DE than in the NDE group (37.9% versus 16.7%; *P *= 0.043; Table 3), due primarily to the emergence of MRSA (27.6% versus 9.5%; *P *= 0.059).

To examine further the association between antimicrobial DE and the emergence of MDR organisms, we performed a multivariable analysis adjusting for potential confounders (Additional file 4). Multivariable-adjusted hazard ratio (HR) for emergence of MRSA was significantly higher with DE than NDE (HR, 3.84; 95% confidence interval (CI), 1.06 to 13.91; *P *= 0.041). The adjusted HR for emergence of MDR Gram-negative rods with DE was lower than NDE (HR, 0.60; 95% CI, 0.17 to 2.07; *P *= 0.418).

## Discussion

This prospective, randomized clinical trial showed that the adequacy of initial antimicrobial therapy is significantly higher with early use of broad-spectrum antimicrobials followed by subsequent DE than with it is conventional therapy. The higher antimicrobial adequacy observed in the DE group was mainly due to MRSA coverage by early administration of vancomycin. However, no significant between-group differences were noted in overall duration of antimicrobial therapy and mortality rates, with the DE group showing a higher rate of MRSA acquisition. Although vancomycin was administered early, the mortality associated with MRSA infections was higher among DE patients. Thus, early administration of vancomycin failed to show therapeutic benefits in our study, and early MRSA coverage before microbiologic documentation seems to be unnecessary.

Early use of broad-spectrum antimicrobials with subsequent de-escalation is considered a promising strategy for optimizing the responsible use of antimicrobials [1]. An observational study of 398 ICU patients with suspected VAP reported that the mortality rate was significantly (*P *= 0.001) lower in patients with DE (17%) than in those with no change in therapy (23.7%) or escalation (42.6%) [5]. That study, however, was observational, with no randomization, and other factors, such as baseline disease severity, may have influenced treatment outcomes, rather than the DE itself.

Previous studies, however, have reported that DE was associated with higher antimicrobial adequacy and more favorable outcomes [6,25-27]. The difference between these results and ours may be attributable to various factors. First, our strategy may have improved antimicrobial adequacy for Gram-positive organisms, most of which were MRSA. Despite increased antimicrobial adequacy of vancomycin against Gram-positive organisms in the DE group, vancomycin had only limited overall efficacy in the treatment of HAP, with no difference in treatment outcomes. Moreover, the mortality rate was higher in our DE than in our NDE group. Vancomycin has been the only antimicrobial available for the treatment of MRSA pneumonia for years [14], but its cure rate has been disappointing [16,17]. Also, despite the broad antimicrobial spectrum of imipenem, its adequacy for Gram-negative organisms was similar in our DE and NDE groups (64.3% versus 85.7%; *P *= 0.19). In a previous study, the adequacy of treatment for Gram-negative organisms was found to be higher in patients treated with a DE regimen, in which the major comparison was between cefotaxime and imipenem, which were used in 39% and 100% of NDE and DE patients, respectively [6]. In contrast, piperacillin/tazobactam (87.3%) and ciprofloxacin (61.8%) were the most frequently used initial antimicrobials in our NDE group. Previous studies have suggested that piperacillin/tazobactam and imipenem/cilastatin have similar efficacy and safety profiles [28-30]. Only one patient had ESBL-producing Enterobacteriaceae, and broad-spectrum antimicrobials may have different sensitivity patterns at other centers. In addition, the definition of "de-escalation" differed among studies, in that most defined DE with regard to Gram-negative organisms. For example, the activity spectrum of antimicrobials was ranked against Gram-negative bacteria, with DE defined as a switch to or discontinuation of a drug class with a less-broad spectrum of coverage [5]. Others have defined de-escalation similarly, by ranking the antimicrobial spectra of these agents [6,31]. In contrast, imipenem/cilastatin and vancomycin were de-escalated individually, with the former de-escalated only in patients with Gram-negative organisms. Categorizing all these definitions by using the single term "de-escalation" is confusing and may result in divergent findings.

The strengths of our study include its prospective design, high initial prevalence of MDR organisms, and relatively high severity of infection. To our knowledge, this is the first randomized clinical trial comparing the DE of imipenem plus vancomycin with that of non-carbapenem, non-vancomycin in a medical ICU. This study, however, had several limitations. First, it was performed in a single center MICU, which may have biased patient selection. The proportion of patients with VAP was relatively small, and carbapenems other than imipenem, such as doripenem, may yield different results. Therefore, the generalizability of this study may be limited. Furthermore, the baseline prevalence of MDR organisms was higher in the DE than in the NDE group, which may have influenced treatment outcomes [32,33]. However, we sought to minimize this effect by excluding these patients from subsequent analyses for emergence of MDR organisms. Moreover, this study was not double-blinded. Indeed, the attending physicians showed a tendency not to de-escalate. Nevertheless, the DE rates were relatively high (83.3% for vancomycin and 84.8% for imipenem/cilastatin). Another limitation was our inability to identify pathogens in 50% of our study patients. The percentage of patients who underwent bronchoalveolar lavage at the time of enrollment was relatively low; thus, our rate of obtaining adequate specimens was not high. A possibility of cross-contamination exists as a source of MDR organisms. We also used the Clinical and Laboratory Standards Institute (CLSI) standards, rather than the European Committee on Antimicrobial Susceptibility Testing (EUCAST) standards to define antimicrobial susceptibility, which may have resulted in a small difference in the incidence of MDR organisms. In addition, our secondary end points, including mortality rates and emergence of MDR organisms, may not have been sufficiently powered to detect significant differences. Also, rectal swabs to identify carriers were not routinely checked. Finally, the dosage of imipenem (2 g/day) may have been insufficient for patients with Pseudomonas pneumonia.

## Conclusions

Although early use of broad-spectrum antimicrobials significantly increased initial antimicrobial adequacy, treatment outcomes were not improved. The therapeutic advantage with early use of broad-spectrum antimicrobials, especially vancomycin, was not evident in this study. Our results should be considered as hypothesis generating, as discussed earlier, and additional clinical research to verify our observations is needed.

## Key messages

♦ Although early use of broad-spectrum antimicrobial agents significantly increased the initial adequacy against multidrug-resistant pathogens among critically ill patients with hospital-acquired pneumonia, it failed to improve survival.

♦ Our data suggest that efficacy of early vancomycin administration is limited, considering that emergence of MRSA in the de-escalation group was even higher than that in the non-de-escalation group.

## Abbreviations

APACHE II score: acute physiology and chronic health evaluation score II; ATS/IDSA: American Thoracic Society/Infectious Diseases Society of America; CLSI: Clinical and Laboratory Standards Institute; CPIS: clinical pulmonary infection score; DE: de-escalation; EUCAST: European Committee on Antimicrobial Susceptibility Testing; HAP: hospital-acquired pneumonia; HR: hazard ratio; ICU: intensive care unit; MDR: multidrug resistant; MRSA: methicillin-resistant *Staphylococcus aureus; *NDE: non-de-escalation; PaO_2_: partial pressure of oxygen; PaO_2_/FiO_2_: ratio of the partial pressure of oxygen to the fraction of inspired oxygen; VAP: ventilator-associated pneumonia; VRE: vancomycin-resistant enterococci; WBC: white blood cell.

## Competing interests

The authors declare that they have no competing interests.

## Authors' contributions

This study was designed by YSK, JC, SBH, and CML. Acquisition of the data was performed by JC, HJJ, SBH, CML, and YK. Analysis and interpretation of data was done by JWK, JC, SHC, and YSK. The manuscript was drafted by JWK, JC, and SHC. Critical revision of the manuscript for important intellectual content was done by SHC and YSK. The statistical analysis was done by JWK and SHC. All authors read and approved the final manuscript.

## Supplementary Material

Additional file 1 **Additional data collection**. Additionally collected data including severity scores and culture specimens. Click here for file

Additional file 2 **Adverse events during the study period**. Adverse events during study period in de-escalation and in non-de-escalation groups. Click here for file

Additional file 3 **Initially identified organisms associated with hospital-acquired pneumonia**. Differences in initially identified organisms in de-escalation and in non-de-escalation groups. Click here for file

Additional file 4 **Unadjusted and multivariable-adjusted analysis for emergence of MDR organisms by using Cox proportional hazards models**. Multivariable Cox proportional hazards models showing emergence of multidrug-resistant organisms in de-escalation and in non-de-escalation groups. Click here for file
