# Supplementary material for: Early use of imipenem/cilastatin and vancomycin followed by de-escalation versus conventional antimicrobials without de-escalation for patients with hospital-acquired pneumonia in a medical ICU: a randomized clinical trial
Source: Crit Care. 2012 Feb 15;16(1):R28. doi: 10.1186/cc11197 (PMC3396273; doi:10.1186/cc11197)
Supplement: Additional file 1 — Additional data collection. Additionally collected data including severity scores and culture specimens. [file cc11197-S1.DOC]

**Additional file 1.** Additional data collection

Two investigators made daily rounds in the medical ICU to identify eligible patients with HAP and to record medical data. Study patients were prospectively followed up from the initial diagnosis of HAP until they were discharged from the hospital or died. For all study patients, the following data were recorded prospectively: age, gender, underlying disease, comorbidities, antimicrobial therapy during the previous 30 days, Acute Physiology and Chronic Health Evaluation (APACHE) II score [1], Sequential Organ Failure Assessment (SOFA) score [2], Clinical Pulmonary Infection score (CPIS) [3], modified McCabe and Jackson score [4], laboratory data, and medications including antimicrobials. Adverse events were monitored during the study period. Microbiological data included all positive respiratory and blood cultures before the administration of study drugs and during the study period. Quantitative or semiquantitative cultures were performed on samples taken from the lower respiratory tract (i.e. sputum, tracheobronchial or bronchoalveolar lavage), and from blood and/or pleural fluid.

**References**

1. Knaus WA, Draper EA, Wagner DP, Zimmerman JE: **APACHE II: a severity of disease classification system**. *Crit Care Med* 1985, **13**:818-829.

2. CDC: **Recommendations for preventing the spread of vancomycin resistance.** *Recommendations for preventing the spread of vancomycin resistance* 1995, **44**:1-13.

3. Pugin J: **Clinical signs and scores for the diagnosis of ventilator-associated pneumonia**. *Minerva Anestesiol* 2002, **68**:261-265.

4. Moise PA, Forrest A, Birmingham MC, Schentag JJ: **The efficacy and safety of linezolid as treatment for Staphylococcus aureus infections in compassionate use patients who are intolerant of, or who have failed to respond to, vancomycin**. *J Antimicrob Chemother* 2002, **50**:1017-1026.
