# Supplementary material for: Early use of imipenem/cilastatin and vancomycin followed by de-escalation versus conventional antimicrobials without de-escalation for patients with hospital-acquired pneumonia in a medical ICU: a randomized clinical trial
Source: Crit Care. 2012 Feb 15;16(1):R28. doi: 10.1186/cc11197 (PMC3396273; doi:10.1186/cc11197)
Supplement: Additional file 2 — Adverse events during the study period. Adverse events during study period in de-escalation and in non-de-escalation groups. [file cc11197-S2.DOC]

**Additional file 2.** Adverse events during the study period

| Adverse events | DE, *n* (%) | NDE, *n* (%) | *P* value |
| --- | --- | --- | --- |
| EPS | 1 (1.9%) | 0 | 0.495 |
| Arrhythmia | 9 (16.7%) | 10 (18.2%) | > 0.999 |
| Cardiac arrest | 0 | 2 (3.6%) | 0.495 |
| Myocardial infarction | 2 (3.7%) | 0 | 0.243 |
| ICU psychosis | 1 (1.9%) | 3 (5.5%) | 0.618 |
| Hypertension | 1 (1.9%) | 4 (7.3%) | 0.363 |
| Rash | 0 | 2 (3.6%) | 0.157 |
| Hepatotoxicity | 0 | 1 (1.8%) | > 0.999 |
| Seizure | 1 (1.9%) | 2 (3.6%) | > 0.999 |
| Thrombocytopenia | 2 (3.7%) | 0 | 0.243 |
| Wheezing | 3 (5.6%) | 3 (5.5%) | > 0.999 |
| Hyperthermia | 8 (14.8%) | 6 (10.9%) | 0.580 |
| GI bleeding | 2 (3.7%) | 0 | 0.243 |
| Diarrhea | 0 | 1 (1.8%) | > 0.999 |

DE, de-escalation group; EPS, extrapyramidal symptoms; GI, gastrointestinal; NDE, non-de-escalation group
