# Supplementary material for: Early use of imipenem/cilastatin and vancomycin followed by de-escalation versus conventional antimicrobials without de-escalation for patients with hospital-acquired pneumonia in a medical ICU: a randomized clinical trial
Source: Crit Care. 2012 Feb 15;16(1):R28. doi: 10.1186/cc11197 (PMC3396273; doi:10.1186/cc11197)
Supplement: Additional file 3 — Initially identified organisms associated with hospital-acquired pneumonia. Differences in initially identified organisms in de-escalation and in non-de-escalation groups. [file cc11197-S3.DOC]

**Additional file 3.** Initially identified organisms associated with hospital-acquired pneumonia

| Organism | DE  (*n* = 53) | NDE  (*n* = 55) | Total  (*n* = 108) | *P* |
| --- | --- | --- | --- | --- |
| Gram-positive | 21 (39.6%) | 15 (27.3%) | 36 (33.3%) | 0.173 |
| *Staphylococcus aureus* |  |  |  |  |
| Methicillin-resistant | 19 (35.8%) | 12 (21.8%) | 31 (28.7%) | 0.107 |
| Methicillin-susceptible | 2 (3.8%) | 3 (5.5%) | 5 (4.6%) | > 0.999 |
| *Streptococcus pneumoniae* | 2 (3.8%) | 0 | 2 (1.9%) | 0.238 |
| Gram-negative | 14 (26.4%) | 15 (27.3%) | 29 (26.9%) | 0.920 |
| *Enterobacteriaceae* | 4 (7.5%) | 8 (14.5%) | 12 (11.1%) | 0.360 |
| *Escherichia coli* | 0 | 3 (5.5%) | 3 (2.8%) | 0.243 |
| *Serratia marcescens* | 1 (1.9%) | 2 (3.6%) | 3 (2.8%) | > 0.999 |
| *Klebsiella pneumoniae* | 2 (3.8%) | 1 (1.8%) | 3 (2.8%) | 0.614 |
| *Enterobacter cloacae* | 0 | 2 (3.6%) | 2 (1.9%) | 0.496 |
| *Enterobacter aerogenes* | 1 (1.9%) | 0 | 1 (0.9%) | 0.491 |
| Non-*Enterobacteriaceae* | 12 (22.6%) | 7 (12.7%) | 19 (17.6%) | 0.176 |
| *Pseudomonas aeruginosa* | 8 (15.1%) | 5 (9.1%) | 13 (12%) | 0.338 |
| imipenem-resistant | 3 (5.7%) | 0 | 3 (2.8%) | 0.115 |
| imipenem-susceptible | 5 (9.4%) | 5 (9.1%) | 10 (9.3%) | > 0.999 |
| *Acinetobacter baumannii* | 3 (5.7%) | 3 (5.5%) | 6 (5.6%) | > 0.999 |
| imipenem-resistant | 1 (1.9%) | 2 (3.6%) | 3 (2.8%) | > 0.999 |
| imipenem-susceptible | 2 (3.8%) | 1 (1.8%) | 3 (2.8%) | 0.614 |
| *S. maltophilia* | 1 (1.9%) | 0 | 1 (0.9%) | 0.491 |
| MDR organism | 24 (45.3%) | 13 (23.6%) | 37 (34.3%) | 0.018 |
| No initial growth (until day 5) | 24 (45.3%) | 30 (54.5%) | 54 (50%) | 0.336 |
| Multiple initial organisms | 7 (13.2%) | 6 (10.9%) | 13 (12%) | 0.714 |

Values given as no. (%) of patients. *P* values by either χ^2^ test or Fisher’s exact test. DE, de-escalation group; MDR, multidrug-resistant; NDE, non-de-escalation group.
