# Supplementary material for: Early use of imipenem/cilastatin and vancomycin followed by de-escalation versus conventional antimicrobials without de-escalation for patients with hospital-acquired pneumonia in a medical ICU: a randomized clinical trial
Source: Crit Care. 2012 Feb 15;16(1):R28. doi: 10.1186/cc11197 (PMC3396273; doi:10.1186/cc11197)
Supplement: Additional file 4 — Unadjusted and multivariable-adjusted analysis for emergence of MDR organisms by using Cox proportional hazards models. Multivariable Cox proportional hazards models showing emergence of multidrug-resistant organisms in de-escalation and in non-de-escalation groups. [file cc11197-S4.DOC]

**Additional file 4.** Unadjusted and multivariable-adjusted analysis for emergence of MDR organisms using Cox proportional hazards models

| MDR organism | Case, *n* (%) | Person-days | Unadjusted | | Multivariable-adjusted^a^ | |
| --- | --- | --- | --- | --- | --- | --- |
|  |  |  | HR (95% CI) | *P* | HR (95% CI) | *P* |
| Overall MDR organisms^b^ |  |  |  |  |  |  |
| NDE | 7/42 (16.7%) | 943 | 1 |  | 1 |  |
| DE | 11/29 (37.9%) | 498 | 2.76 (1.07−7.16) | 0.036 | 2.69 (1.01−7.22) | 0.049 |
| MRSA^b^ |  |  |  |  |  |  |
| NDE | 4/42 (9.5%) | 976 | 1 |  | 1 |  |
| DE | 8/29 (27.6%) | 528 | 3.47 (1.04−11.56) | 0.043 | 3.84 (1.06−13.91) | 0.041 |
| MDR Gram-negative rods^b^ |  |  |  |  |  |  |
| NDE | 5/42 (11.9%) | 973 | 1 |  | 1 |  |
| DE | 4/29 (13.8%) | 602 | 0.64 (0.19−2.19) | 0.478 | 0.60 (0.17−2.07) | 0.418 |

CI, confidence interval; DE, de-escalation group; MDR, multidrug-resistant; MRSA, methicillin-resistant *Staphylococcus aureus*; NDE, non-de-escalation group; HR, hazard ratio.

^a^ Adjusted for age, sex and APACHE II score

^b^ Excluding patients from whom MDR organisms had been initially isolated.
